# Experiences of using surveillance cameras as a monitoring solution at nursing homes: The eldercare personnel’s perspectives

**DOI:** 10.1186/s12913-023-09130-2

**Published:** 2023-02-10

**Authors:** Maria Emilsson, Christina Karlsson, Ann Svensson

**Affiliations:** 1 Department of Health sciences, Section of Nursing Graduate Level, University West, SE-461 86 Trollhättan, Sweden; 2 Department of Health sciences, Section of Nursing Undergraduate Level, University West, SE-461 86 Trollhättan, Sweden; 3 School of Business, Economics and IT, Division of Informatics, University West, SE-461 86 Trollhättan, Sweden

**Keywords:** Surveillance camera, Nursing home, Eldercare personnel, Privacy, Older people

## Abstract

**Background:**

As the number of older people increases, so does the need for care. However, the workforce in eldercare cannot increase at the rate required to match the needs. Welfare technologies, such as surveillance cameras, can replace physical visits and be used at night to monitor older people in order to keep them safe, while not disturbing their sleep. The aim of the paper is to analyze obstacles and opportunities associated with implementation and use of surveillance cameras at nursing homes from the perspectives of the practitioners who use the technology, their working environment and the conditions of the older people with cognitive impairment who live in nursing homes.

**Methods:**

Individual semi-structured interviews were conducted with the eldercare personnel at nursing homes to understand their experiences of implementation and use of surveillance cameras. The transcribed interviews were analyzed using qualitative content analysis. The consolidated criteria for reporting qualitative research (COREQ) was used as a guidance tool.

**Results:**

The results show that the eldercare personnel experienced lack of adequate information, education and support related to using surveillance cameras. Several benefits are highlighted, such as better working environment and that the residents were not unnecessarily disturbed at night. However, the results also show that it is important to clarify that surveillance cameras cannot replace the human presence.

**Conclusions:**

The conclusions from this study are the importance of prerequisites for implementation, and that using surveillance cameras contributed to improvements in the working environment at night and created possibilities to maintain security and integrity for older people living in nursing homes.

## Background

In the Nordic countries, surveillance cameras are widely recognized as a type of welfare technology that can be used as support in the care of elderly people [1]. Related to health and well-being, welfare technology can be used at a distance or at the physical place. Such technologies have the potential to provide high-quality care services to older people, and relieve eldercare personnel, as their human hands are a scarce resource in the society [2]. Experiences show that the use of welfare technologies offers support to residents and eldercare personnel [3]. Consequently, there is an increasing demand to develop social care services that utilize welfare technologies [4]. According to the World Health Organization (WHO), the population grows older worldwide [5]. As the number of older people increases, so does the demand for care with sustainable quality [6, 7]. The number of people working in eldercare cannot increase at the rate required to match the needs for care, as the number of older people increases. WHO estimates that the share of the global population that is 60 years or older will increase from 12 to 22% between 2015 and 2050 [5]. The growing number of older people will live in nursing homes or alone in their own private homes. Many older people need support to be able to live at home as long as possible, when they can no longer take care of themselves due to age or illness.

Monitoring solutions, such as surveillance cameras, are used to remotely monitor older people’s activity, health status and safety in their rooms at nursing homes or in their own private homes [8]. In this paper, we define a nursing home as long-term care home for older people in need of service, assistance, and care around the clock and on a daily basis. Nursing homes offer all the support that the residents need in everyday life, including healthcare interventions by nurses, physiotherapists as well as visits by doctors. Such technology, with digital surveillance at a distance, can replace physical visits and preferably be used to monitor older people at night in order to keep them safe, while not disturbing their sleep [3, 9]. Such surveillance cameras can sometimes be used together with activity monitoring alarms close to the bed, in order to reduce the number of physical visits at night [9]. When an alarm is activated, the eldercare personnel do not always need to physically visit the older person. Instead, they can use the surveillance camera to check the status at a distance. This way of providing social care can increase the privacy and safety of older people. According to Richardson et al. [10], there is a need for more high-quality intervention studies to gather evidence of the benefits of nocturnal digital surveillance technologies. Eldercare personnel experience lack of evidence of the benefits of the technology, although some projects have been running for a long time.

Some studies in this field focus on different privacy issues, by, for example proposing solutions to data security threats, while other studies focus on the benefits of using surveillance devices. Family members as well as residents emphasize that privacy invasion is an important issue [11]. Using camera-based surveillance systems is considered to increase the risk for privacy invasion compared to non-camera-based systems. For this reason, it is important to find the right balance between increased safety and decreased privacy, when using surveillance [12]. Alkhatib et al. [8] state the importance of conducting empirical studies to further explore privacy issues related to the use of surveillance devices, as well as other limitations of such technologies. Moreover, Azimi et al. [4] call for user-centered studies that focus on capabilities, requirements, and abilities of the users. When implementing technology in the care of older people, ethical issues must be considered [13]. Other aspects associated with using surveillance devices that also need to be considered include user experiences, the roles and actions of the eldercare personnel, and the effects on the quality of the older people’s life [14].

The aim of this paper is to analyze obstacles and opportunities associated with implementation and use of surveillance cameras at nursing homes from the perspectives of the eldercare personnel who use the technology, their working environments, and the conditions of the older people with cognitive impairment who live in nursing homes.

The paper is outlined as follows; the second section describes the theoretical background of the use of surveillance cameras; the third section describes the empirical setting and the research method; the fourth section presents the results; the fifth section discusses the results, and the sixth and final section presents the conclusions of the paper.

### Theoretical background of the use of surveillance cameras

Advancements in welfare technology have enabled increased functionality and more enhanced and efficient services for older people, regardless of whether they live in a nursing home or their own private home. Video monitoring of residents at nursing homes via surveillance cameras is an advancing welfare technology that can assist healthcare organizations in meeting operational demands and providing safe optimal care [15].

The willingness to use welfare technology is higher if the residents feel more secure with the technological devices. However, it should be noted that not everyone wants a camera in their room [16, 17].

The residents’ privacy at the nursing home is of vital importance, and there must be zero tolerance for any abuse. According to Berridge et al. [18], we need to consider the ethical aspects of using surveillance cameras more, as well as the perspectives of various stakeholders. It is important that the workforce is well-trained, as they need to know how to maintain the hardware and software of the surveillance equipment [16]. The older people’s individual needs have to be taken into account when using surveillance cameras, to keep people safe [14]. In this way, surveillance cameras can offer reassurance to people who might be scared of falling, since the eldercare personnel can monitor their activities and conditions at a distance. Nevertheless, it can be difficult to understand what is legal and lawful when using welfare technologies [3]. The nursing homes need to take responsibility for the maintenance and proper working of the surveillance cameras.

Nursing homes need to obtain informed consent from the residents who could benefit from surveillance cameras in their rooms, while taking their cognitive capacities into account. If an older person suffers from cognitive impairment, family members or other appropriate persons should be involved in the decision. Family members, relatives and other people who visit nursing homes need to be informed about how and when surveillance cameras are used, and how the videos are handled [14]. Family members of the residents seem to appreciate the surveillance technology, with features such as location tracking, in-home activity sensors, and 24-h web cameras, more than the residents. Their opinions differed the most when it came to web cameras [11]. The eldercare personnel at the nursing homes have certain responsibilities regarding the use of surveillance cameras, and there are predefined rules that they have to follow, for example regarding how long time an older person may be monitored at a time, and how many times an older person may be monitored during a defined time period. The surveillance cameras also have to be clearly visible. Moreover, the eldercare personnel’s conduct is monitored and recorded, to ensure safe and ethical use of surveillance cameras from the residents’ point of view, which contributes to a good and more efficient care practice for the eldercare personnel [9].

## Methods

### Research context

The use of surveillance cameras was studied at three nursing homes in a small, sparsely populated municipality in Sweden, where the majority of the residents had cognitive impairment. About 50 residents lived in the nursing homes, of which 44 had cameras during the study. Surveillance cameras had been offered to those residents who had been judged to be able to benefit from it, and been installed in their rooms after obtaining their written consent. The older people living in the nursing homes and their relatives had received both oral and written information about the use of surveillance cameras.

### Research design

The aim of this paper is to analyze obstacles and opportunities associated with implementation and use of surveillance cameras at nursing homes from the perspectives of the eldercare personnel who use the technology, their working environments, and the conditions of the older people with cognitive impairment who live in nursing homes. The study was conducted six months after the introduction of the surveillance cameras and focuses on whether the residents are helped by the use of surveillance cameras in their rooms, as a complement to ordinary night surveillance, and how the eldercare personnel experience the use and usefulness of the surveillance cameras.

The participants in the study received both oral and written information about the study and the reasons for conducting the research. First, the nursing home manager informed about the study and asked the residents to participate. At the time of the interviews, the researchers presented themselves and their roles as researchers, roles in the study and in the broader context of the ongoing project. The researchers also orally presented the aim of the study.

### Research approach

An inductive approach was used in this case study [19]. The consolidated criteria for reporting qualitative research (COREQ) was used as a guidance tool [20]. A literature review within the area of use of surveillance cameras was carried out using Google Scholar. The most important keywords were: surveillance camera, older people, residents, relatives and nurses. The case study produces context-dependent knowledge and experiences of the use of surveillance cameras at nursing homes [21]. The interpretations and reflections of the results are compared with the existing literature. The research is influenced by the interpretive perspectives as it focuses on understanding the context and the processes of human sense-making in monitoring older people with dementia with surveillance cameras at nursing homes [22]. One gap in the existing research as identified in the literature review is the lack of evidence of the benefits of the technology within this area.

### Data collection

The data were collected by semi-structured interviews [23, 24]. The questions were created by the researchers AS and CK. We asked the participants to describe how they worked before surveillance cameras were introduced, how training for using the surveillance cameras was arranged, and how a work shift might look like with surveillance cameras as a supplement. We also asked about the advantages and disadvantages of surveillance cameras.

The interviews were conducted at the end of 2019, just before the pandemic began. The nursing home manager asked the night shift staff to participate in the study, as a purposive selection of participants. All staff members who worked night shifts were interviewed; in total twelve people. Staff members who worked nights were directly affected by the use of surveillance cameras. Some day-time staff were also asked to participate, in order to catch effects that emerged during the day. Three of the interviewees worked day shifts, and hence had experience of how the use of surveillance cameras affected the residents during the day, when they were awake. A unit manager (a nurse), was also interviewed. The manager did not directly use surveillance cameras in her work, but she had an overview of the experiences of using cameras at the nursing home. All participants except the unit manager were assistant nurses. Everyone who was asked to participate in the study agreed to do so, and everyone signed a written informed consent. A total of 16 people from the eldercare personnel were interviewed, see Table 1. No one refused to participate.

**Table 1:** Interviewees in the study

Interviewee	Working years in eldercare	Role during the study
IP 1	14	Night shift
IP 2	30(of which 10 as a manager)	Manager
IP 3	35	Night shift
IP 4	30	Day shift
IP 5	34	Night shift
IP 6	32	Day shift, technical knowledge
IP 7	12	Night shift
IP 8	20	Night shift
IP 9	20	Night shift
IP 10	8	Night shift
IP 11	6	Night shift
IP 12	22	Night shift
IP 13	27	Night shift
IP 14	5	Night shift
IP 15	25	Night shift
IP 16	5	Day shift

The participants were interviewed in a room at the working place or by telephone from home, when they were not working. Only the participants and the researchers were present at the interviews. The researchers who conducted the interviews (Professor AS and Research assistant CK) have experience in conducting qualitative interviews. The researchers (AS and CK) considered the interview responses clear, hence no interview had to be repeated. At the time of the interviews, the participants had worked on average 20 years in eldercare, with a minimum experience of five years. This level of experience could be considered an important characteristic of the participants in the study. The data collection focused on acquiring a deep insight and understanding of how the eldercare personnel experienced the use of surveillance cameras and how it affected the monitoring conditions at night at the nursing homes. The interviews were semi-structured, and each interview lasted between 15 and 45 min. All interviews were audio recorded and transcribed verbatim. Notes were also taken during the interviews. The transcripts were not returned to the participants for comments and/or corrections, but the participants were invited to contact the researchers if they had questions or comments relating to the interviews or their personal data. A written summary of the study has been provided to the nursing home, via the manager.

### Data analysis

The interviews were inductively coded (without software) in order to contribute to existing research objectively, through “close adherence to data” [25]. The researchers (AS and CK) read the transcripts multiple times to gain a deep understanding of the data. Categories of the data should be able to describe and explain the phenomenon [26] and link the findings to the aim of the research [27]. The e content analysis [26] was done iteratively by two of the researchers (AS and CK) in a circulative process that aimed at developing codes and categories based on the data. A critical analysis and reflection of interpretations was conducted, before going back to analyzing the data again. Quotes that strengthen and illustrate the statements in the results were identified in the transcriptions. This procedure was conducted to enhance the rigor of the research [27]. One more researcher (PhD ME) joined the research team in the final phase of the study, which focused on deepening the analysis, further discussing the interpretations of the data, codes, and categories; comparing them with the existing research literature, and reporting the research findings. An example of meaning units and how they have been interpreted as sub-themes and main themes is presented in Table 2. All three researchers (AS, CK and ME) have worked together in previous research studies.

**Table 2:** Meaning units, sub-themes and main theme

Meaning unit	Sub-theme	Main theme
We are one in each department, so I check [the cameras] where I am, but we also help each other when it’s needed. We can also check for each other, if you are working in a room and can’t leave, but the colleague knows it, so it’s… We help each other a lot. IP 1	Easier to help each other	Improvements in the working environment at night
You can prioritize in a completely different way when you have cameras, because then you can see. If there are several bed alarms at the same time, for example, then you can see, ‘no, that was just the blanket, but there, Greta is up’. Then you can run to her, rather than to run and wake up the person where it was just the blanket and get him up too. IP 1	Easier to prioritize	
We don’t go into the room if we don’t have to, because there is a lot of banging on the doors, so we look at the camera. IP 4	Easier to prioritize	

### Ethical statement

The study has been approved by the Swedish Ethical Review Authority (Approval number: 932–18), and the Helsinki Declaration has been taken into account [28].

## Results

The study resulted in three main themes and seven sub-themes, see Table 3. The main themes are: 1) Prerequisites for implementation, 2) Improvements in the working environment at night, and 3) Possibilities to maintain security and integrity.

**Table 3:** The main themes and the sub-themes that emerged from the inductive content analysis

Main themes	Sub-themes
Prerequisites for implementation	Need for adequate information and education Importance of functionality Need for support
Improvements in the working environment at night	Easier to help each other Easier to prioritize
Balance between security and integrity	Ability to not disturb unnecessarily Not a substitute for human presence
	Importance of integrity

### Prerequisites for implementation

Prerequisites for implementation of surveillance cameras in connection with the start-up is considered as a main theme. The theme highlights the need for adequate information and education, the importance of the functionality of the cameras, and that knowledgeable support should be available.

### Need for adequate information and education

The eldercare personnel were given information about how the surveillance cameras worked in the beginning of the study, but it was insufficient when problems arose during the installation. They wished that they had more information about how the cameras should be used beforehand, and how their working methods would change.

The eldercare personnel received training in handling the surveillance cameras for two to three hours in connection to the installation of the cameras by the supplier. During the installation, however, functionality problems arose, which caused the cameras to remain unused for several months. Hence, the information and training were provided too early, long before the eldercare personnel actually started using the cameras.

*There was an education, but it* [the delivery of the cameras] *became so delayed, because they said that we would get them in December, I think… so it took a very long time, because it was in March or something. It took such a long time before the cameras came. Wait three months. You have forgotten again.* IP 3

*No, no education. We just went through how it worked at the introduction, so no training other than that.* IP 14

All eldercare personnel were not able to participate in the training.

*There are some, who have received education who are a little more responsible for it* [the technology] *and so, but the rest of us had to learn from them.* IP 13

The eldercare personnel considered that the company representative who provided the training had insufficient knowledge about the functionality of the surveillance cameras. Also, when the eldercare personnel contacted the company’s support function, they did not get sufficient help. In retrospect, they wished that they had put higher demands on the company to come to the nursing homes and solve the problems from the beginning.

*I don’t think the staff at the company that has the cameras really knew how to do.* IP 9

The insufficient knowledge about how the surveillance cameras worked resulted in the eldercare personnel initially feeling insecure. For example, they thought that the cameras were recording all the time, and that they were filmed during their work shifts (they later learned that this was not the case). Some members of the staff felt uncomfortable being on film, and with the idea that someone could sit and watch them. When the cameras did not work, the eldercare personnel initially thought they had done something wrong.

*It was a bit difficult to understand how it should work, kind of. One* [colleague] *thought that the cameras were on all the time. Well, shall we be there then? Some felt uncomfortable that we should maybe be on film, did someone sit and watch us? We joked about it, that we must look good in the hair before going to work.* IP 3

The eldercare personnel were also unsure if the cameras had any audio function, because they could sometimes see a speaker icon on the screen of some cameras, but they never heard any sound.

*There may be sound, but I have never really got a grip of it, because sometimes you see on the screen that there is such a speaker icon, or thing. But not on all of them, and I have never really heard any sound, so I actually don’t know how it works.* IP 8

The eldercare personnel experienced that the training seemed to be good while they participated in it, but afterwards, they realized that it did not correspond well with the practical use. The information they received did not work in reality, and there were major problems when they turned on the cameras, because of incorrect information. Hence, they had to figure out how to proceed themselves. One of the staff members who worked during the night, and who had technical knowledge, prepared simple instructions for her colleagues to use. She also had shortcuts set up on the computers at the departments to facilitate access to the software. After a while, the eldercare personnel found it easy to understand how to use the surveillance cameras. They considered them not so complicated to use, once they had learned it.

*First, we got the wrong instructions on how to do, so I probably sat for several hours and had to figure out how to solve the problems*. IP 15

*When you have learned it, it’s not difficult.* IP 5

### Importance of functionality

The functionality of the surveillance cameras was important, but the eldercare personnel faced some problems. For example, the image could suddenly disappear. It took time for the eldercare personnel to try to solve such problems, which, however, diminished over time. Some cameras had to be replaced due to blurry and foggy images. The eldercare personnel also experienced problems with cameras that got stuck “chewing”, and had to be restarted. Other cameras took a long time to start or had poor image resolution.

*I think it takes a little too long before the camera connects, if you say so. It stands and chews a little long, you think sometimes*. IP 5

The cameras had to be turned on manually via the computer early during the night shift at a given time interval, in order to work for surveillance during the night. If not, the cameras did not work during the night. This surprised the eldercare personnel, as they thought that the cameras would start automatically. Therefore, the healthcare personnel created a routine to start all cameras between 9 and 11 pm, during the night shift. Previously, the start-up time had been between 10 and 11 pm, but it changed after six months.

*Now we live like in 2019, if I have a camera in a room, then I think that when the time is 21:00, that camera should be on, can I in my little world think. I should not have to go in and activate it.* IP 8

Inspection via camera could be done quickly, to determine the cause of an alarm, for example. Sometimes, however, the eldercare personnel experienced that the cameras started too slowly, which forced them to run to the resident’s room for inspection. When camera images were delayed for too long, safety and security was reduced. The eldercare personnel therefore wished to have faster camera connections.

*It can take a while before it’s connected, sometimes then it’s better to go and check.* IP 11

*Well, when it works, it's very good, but it's been a bit of a hassle and some nights the camera hasn't worked. And then you notice a lot how quickly you get used to the camera, how good you think it is, once they are gone just one night. If they work, they are great.* IP 1

The surveillance cameras were placed in the residents’ rooms so that the eldercare personnel could see the residents when they were in bed, via a computer in the staff room. The cameras were not optimally placed in all rooms, as some residents refurnished their rooms. Generally, the cameras had a relatively good range inside the rooms, which made it possible to see if the residents were in bed. However, the surveillance cameras did not reach the toilet. Each department only had one computer, placed in the staff room, where the eldercare personnel could watch the videos from the surveillance cameras.

*They may not fit so well in all rooms. But then it's like that, you set up a camera and then the patient wants the bed in another place*. IP 8

The eldercare personnel had tried to watch the surveillance videos via their mobile phones, as had been intended from the beginning, but it had not worked. Being able to use the mobile phone would have made it even easier, since the healthcare personnel would not have to run to the staff room to watch the videos via the computer, at each alarm.

*Something that you can press so you can see them directly in the camera in the mobile, if you are… It must be good for them at home care when they are out and about and maybe have them* [their mobile phones] *with them*. IP 6

### Need for support

The eldercare personnel were referred to different people, who gave different answers, each time they contacted the support function. Moreover, the supplier offered no support at night, when it was mostly needed. The municipality’s IT department and the eldercare personnel at the nursing homes had to solve many problems themselves, which took a lot of time. One of the staff members, who was technically knowledgeable, contacted the supplier of the surveillance cameras and addressed the problems that arose, and corrected things that went wrong.

*So, then I got help via support on how to do, even though there was a manual, but it wasn’t... there were lots of different ways to do and we didn’t get any information about that either, but we had to learn it ourselves, quite simply. I think I lived on the phone, more or less, to get it started.* IP 16

*So, you are only referred to a support and there are different people, and they respond differently.* IP 2

If the eldercare personnel had known how badly it would work from the supplier’s side, regarding training and support, they would have prepared the introduction in a completely different way. They would have tested more, with only one camera from the beginning. They would also have more firmly demanded that the supplier came to the nursing homes and solved the problems.

*If I had known how badly it would work from the company, I would have done it in a completely different way. Then we had probably, before we sort of... started, yes, tested more on someone, like this test patient who does not exist, and then tried to technically solve it.* IP 2

### Improvements in the working environment at night

When the surveillance cameras worked, they facilitated the work during the night by making it easier for the eldercare personnel to help each other across departments, and prioritizing who to attend to first, when there were several alarms at the same time.

### Easier to help each other

The eldercare personnel generally found that the cameras made the work at night easier, since their colleagues in other departments could help them watch the films from their cameras. One person usually worked alone in each department at night, but the surveillance cameras made the eldercare personnel feel that they could work together, across departments. When there were multiple alarms at the same time in the same department, colleagues from other departments could come over and help respond to the alarms. The eldercare personnel considered that this benefitted the residents, by reducing disquiet moments.

*We are one in each department, so I check* [the cameras] *where I am, but we also help each other when it’s needed. We can also check for each other, if you are working in a room and can’t leave, but the colleague knows it, so it’s... We help each other a lot.* IP 1

Increased cooperation between eldercare personnel who helped each other with alarms meant that the residents received help more quickly, which in turn meant increased safety and security for the residents at night.

### Easier to prioritize

The eldercare personnel could see why an alarm had been activated in the system, via the camera. The reason could be, for example, that the resident was on his/her way out of bed, that part of the blanket had fallen down, or that the resident had moved in bed. The eldercare personnel could also see via the camera if an arm or a leg protruded outside the bed, and assess if it was necessary to enter the resident’s room or not. The staff thus used the surveillance cameras to assess the need for help when bed alarms had been activated. All in all, the cameras contributed to less work effort for the eldercare personnel and enabled them to make priorities in a completely different way, compared to during physical visits.

*You can prioritize in a completely different way when you have cameras, because then you can see. If there are several bed alarms at the same time, for example, then you can see, ‘no, that was just the blanket, but there, Greta is up’. Then you can run to her, rather than to run and wake up the person where it was just the blanket and get him up too.* IP 1

*We don’t go into the room if we don’t have to, because there is a lot of banging on the doors, so we look at the camera.* IP 4

When there were several alarms at the same time, the eldercare personnel could prioritize who to go to first and who did not need a visit, so-called false alarms. Fall-prone persons on their way out of bed were prioritized first. If the eldercare personnel knew that a resident was prone to falls and usually got out of bed quickly, they went straight to the room, without first looking via the camera. This provided greater security and safety for both residents and eldercare personnel.

*Some we don’t go to at all, we only look at them in the camera, some we go to, yes, it’s different from person to person. If a person is anxious and you know it, then I look in the camera first, and enter afterwards.* IP 9

### Balance between security and integrity

By using the surveillance cameras to prioritize who to help first and who could do without supervision, the eldercare personnel avoided disturbing the residents unnecessarily, which led to calmer nights for the residents. The eldercare personnel considered it important to maintain the residents’ integrity, but found that the surveillance cameras could not substitute human care.

### Ability to not disturb unnecessarily

The eldercare personnel knew in advance for which residents and in which situations they could look via the camera before entering the room, as they got to know the residents and adapted their routines to them. If they saw that a resident who often needed help was up, perhaps after a visit to the bathroom, they often went to the resident’s room and helped the resident with, for example, getting back in bed. Alternatively, they looked via the camera again after a while, to make sure that the resident was back in bed. Some residents were up walking in their rooms at night, and when the eldercare personnel could use the camera to see that everything was in order, they did not have to go in and disturb them.

*You don’t need to disturb those who don’t need to be disturbed, when you have the camera. When alarms go off and things like that. It feels like they get a better night, if you say so.* IP 5

The eldercare personnel had an ordinary routine of monitoring the residents who needed it and wanted it, several times a night. It was, however, difficult to monitor quietly. Many residents woke up easily, and woke up every time a member of the staff entered their room for monitoring, due to noise from the door. The residents were sometimes also awakened by the activation of other residents’ bed alarms, since the eldercare personnel carried alarm phones in their pockets.

*Yes, that's what's so nice, if they are worried and easily awakened… maybe they are sleeping and it’s just the blanket or the comforter or the hand that is down, if someone enters then, opens the door, a new alarm may be activated – then they may wake up. Instead check the camera and see: they are sleeping, they just moved a bit.* IP 3

The eldercare personnel usually woke up the residents at least three times per night, which for natural reasons negatively affected their night’s sleep. It could also lead to inability to rest and fall asleep again, as well as increased anxiety and agitation. Sleeping pills or sedatives was sometimes a necessary measure.

*It’s a good night's sleep, less sedative, less sleeping pills, it’s that they get more privacy even though they live in a nursing home. Yes, it's like at my home, for example: I don’t want people to come and check on me three times per night and sometimes more. Then it would have been better if they check on me in a camera if everything was fine, so they don’t have to come, if it’s calm. And if I need help, it can be seen there.* IP 1

Some residents had sensor-based bed alarms with motion sensors that activated a sound alarm, if, for example, the resident attempted to leave the bed. Sensor-based bed alarms were also activated if part of the body, such as an arm or a leg, pointed outside the bed. Primarily people with an increased risk of falling, or who were not able to find their way to the toilet, had such bed alarms. Bed alarms that had been activated had to be checked by the eldercare personnel, which could happen several times per night. This led to frequent monitoring that risked awakening the residents, if the personnel physically entered the rooms.

To avoid disturbing the residents’ sleep, the eldercare personnel used the cameras during the regular rounds at night, and when bed alarms had been activated, instead of physically entering the residents’ rooms. In this way, the residents who were asleep could continue to sleep without being disturbed. This was also reflected during the day, as the residents became more tired if they had not been able to sleep properly during the night.

Residents who could not sleep at night because they had been disturbed, instead slept during the day, or became anxious and then needed more sedatives during the day. Especially in people with cognitive impairment, the brain gets tired easily, and fatigue creates anxiety.

Residents usually woke up every time the eldercare personnel entered their room for monitoring, which in turn triggered the need to urinate. Physically monitoring the rooms thus led to increased use of incontinence aids.

*Every time you go in and check on them and wake them up, then I know, I have seen a study, that if you wake up, you pee, so then there is an increased use of incontinence aids, that you have to change*. IP 2

Reconciliation team meetings were organized and attended by a nurse, staff from rehabilitation, as well as staff working both day and night shifts at the nursing homes. One point on the agenda was how the resident sleeps at night. The manager claimed that the staff more often said that the residents slept well at night, when surveillance cameras were used, and that they became less worried and anxious. The residents sometimes had different sleep patterns. The eldercare personnel also experienced that the residents slept longer during the night when surveillance cameras were used.

*And if you sleep better at night, there is a greater chance that you don’t have anxiety during the day because you are tired, the brain is exhausted, and it’s easily exhausted in a dementia patient. Then you have less anxiety during the day, so less use of sedative drugs*. IP 2

### Not a substitute for human presence

The eldercare personnel did not consider the surveillance cameras to be enough. Sometimes they still had to visit the residents in their rooms. Especially when residents were ill or felt unwell, they naturally went into the rooms more often. Cameras do not offer the possibility to smell, feel the body temperature, see the face color, hear the breathing, and so on. An advantage with physical monitoring was that the eldercare personnel could use all their senses. Sometimes only the human presence helped against anxiety. A warming hug, for example, can never be replaced by a camera.

*The downside is that you can enter in the morning and maybe… well, with the camera you don’t see feces. They may be in stool. We have entered and there has been vomit. You don’t see that with the camera.* IP 15

*You still have to check, so they haven’t fouled themselves maybe and lie in feces, and such things. You have to keep track of it. And you can’t do that through a camera.* IP 5

### Importance of integrity

The residents did not seem to be disturbed by the surveillance cameras in their rooms, according to the eldercare personnel. Only on a few occasions did the eldercare personnel experience that not all residents were completely comfortable with having surveillance cameras in their rooms.

*It is often enough to look at the camera to see that the patient is ok. Some want to fend for themselves, and some, they get a little offended when you come in. Then it can be good to have that camera and look at as well, and know that everything is under control.* IP 11

Physical monitoring with staff who enter the residents’ rooms can be perceived as more intrusive than digital monitoring via cameras. Not all residents wanted staff to come and check on them. The residents often felt that the eldercare personnel followed them, or looked at them, which made them feel monitored and watched. The residents expressed that it affected their privacy and integrity, when personnel entered their rooms.

*No, I don’t meet a lot of relatives, but I know someone who has hung a cap over it* [the camera], *for example, because they feel monitored, and that’s a bit sad.* IP 9

*It could be that someone might feel monitored, or well, but not a lot. But that's how it is maybe, I know someone who’s very suspicious of most things. It would probably absolutely not be possible to put a camera in their room. Because it could turn into something very negative. And that's a bit of a shame, because it could also be the kind of person who could really benefit from a camera.* IP1

To avoid misunderstandings, it was important that both the residents and their relatives received detailed information about how the surveillance cameras worked and how they were used. The eldercare personnel expressed that the residents and their relatives had to be clearly informed that the cameras were used with satisfactory safety. A logbook was kept with information about which members of the staff had watched the films from the cameras, and when. The watching time was also limited to a few minutes per session, after which it was only possible to get another 30 s.

## Discussion

The eldercare personnel experienced that the nocturnal digital surveillance technology had several benefits for the residents, such as giving them better night’s sleep, reducing the use of sedatives and sleeping pills, and making them more alert during the day. The cameras also facilitated the eldercare personnel’s work by making it easier for them to help each other across departments, prioritize, and create a better work environment. These findings contribute with information about the benefits of nocturnal digital surveillance technology, reducing the previous lack thereof [3, 10]. The results from this study also make it possible to give useful feedback to the eldercare personnel. This study is one of the first to report results from an intervention of this kind, and what can be learned from it, and hence contributes to filling a gap in the existing research literature [29].

Initially, the eldercare personnel felt insecure about how the surveillance cameras worked. Better information could have prevented misunderstandings about the function of the cameras, and that the eldercare personnel felt uncomfortable before the upcoming intervention. It was emphasized that not everyone had the opportunity to participate in the introduction, and that the time between the training and the implementation was far too long, about four months. The eldercare personnel hence felt that they were unprepared before the implementation of the surveillance cameras. Similar findings are reported by Glomsås et al. [30]. Involving everyone in an intervention from the beginning may ensure that each working shift has knowledge about the new technology, and that everyone feels involved. The eldercare personnel felt they were not involved in the implementation of the surveillance cameras, and they were concerned that it negatively affected the care of the elderly, which can be confirmed by Glomsås et al. [30]. Not being involved can make the eldercare personnel feel left out [29], which in turn may lead to a lack of interest in learning to use new welfare technology.

In order to gain confidence and develop positive feelings about welfare technology, it is important to first get adequate information and build competence, before starting to use the new technology [30]. One of the staff members had technical knowledge and created user instructions for her colleagues. She also had shortcuts set up on the desktops of the computers. This made it easy for the eldercare personnel to understand how to use the surveillance cameras. Other research also points out the absence of written instructions and explicit routines as a barrier [31].

The eldercare personnel expressed that the supplier should have given the information in a different way, when they needed support and help with the functionality. The information given to the eldercare personnel could not be fully applied because it did not match the practical use.

Including the eldercare personnel in the development of the functionality of the surveillance cameras, and the related working methods, could have prevented two issues; first, the perception of incorrect information, and second, the experience of technical failures. Small enterprises have in previous research expressed that healthcare personnel should be involved in the development of welfare technologies for primary healthcare applications, in order to ensure that the technologies fit the contexts in which they are going be used [32]. This finding is transferable to other healthcare sectors, such as eldercare.

The eldercare personnel experienced major problems with the cameras because of incorrect information, as well as lack of information. Hence, they had to figure out for themselves how to proceed. This is in line with the results from Dugstad et al. [31], who concluded that problems with the equipment represented the biggest challenge in the beginning. Similar results emerged in another Norwegian study [30]. Language barriers may be another reason why the information could not be transferred to the practical context. Language barriers between healthcare personnel and technicians have previously been reported to hinder knowledge integration, as well as learning and motivation to use new welfare technologies [31, 33].

It is important that the older people living in nursing homes and their relatives get detailed information about how the surveillance cameras work and how they are used. The residents and their relatives need to know that the cameras are used with satisfactory safety. In order to be able to provide adequate information, the eldercare personnel need to have knowledge about the technology [30]. Ethical dilemmas can arise when consent is needed from a person with cognitive impairment. Even if the person receives adequate information and understands it at that moment, the person may forget it shortly afterwards. A surveillance camera installed in a person’s room can therefore be perceived negatively even though the person has approved it before. Therefore, people with cognitive impairment who have been offered a surveillance camera in their room need to be subject to an individual continuous assessment.

The work environment improved in some ways after the cameras were taken into use. Normally, one person from the eldercare personnel worked alone in each department at night. Thanks to the cameras, they could start to help each other across departments, by looking at each other’s cameras. The cameras also helped the eldercare personnel prioritize who to help first. Thanks to the cameras, the eldercare personnel did not need to go on observation rounds to see if the residents needed help. Similar results were found in the study by Geil Kollerup et al. [34]. Fewer observation rounds implied that the older people were less disturbed while sleeping. The eldercare personnel used the cameras to see that everything was in order with the residents. Thanks to the technology, they did not need to enter the rooms and disturb the residents at night. Hence, less sedatives were used, and the residents could sleep better.

Bringing a surveillance camera into a person’s home should be preceded by discussions about possible privacy implications. The possible benefits should be weighed against the possible negative effects of the camera. Comiskey et al. [35] asked caregivers in the planning phase about their opinions about installing cameras for monitoring their relatives in need of care at home. The caregivers expressed concerns about privacy for their relatives.

Privacy is of vital importance and there must be zero tolerance for any abuse [16]. Having clear guidelines in place may avoid irregularities such as invasion of privacy and misuse. The guidelines should be explicit and everyone in the eldercare personnel should know them, including hourly employees. Such guidelines may include information about where the cameras cannot be placed. The cameras should, for example, not reach the bathrooms. Likewise, ethical issues must be considered before using cameras, as noted by Sundgren et al. [13]. To prevent ethical problems, the eldercare personnel also need to know how to maintain the hardware and the software [16]. Fulfillment of such requirements suggests that surveillance cameras may be ethical as they deliver care services with a higher autonomy for the older people [36].

A downside to the efficiency of surveillance cameras is the risk for abuse. In the event of staff shortages and budget cuts, cameras could become a tool that is used to replace staff. However, nocturnal digital surveillance technology cannot fully replace the human presence. The eldercare personnel expressed that they still needed to visit residents who were ill or felt unwell. Humans also need to be physically present to smell, feel the body temperature, see the face color, and hear the breathing. Likewise, physical contact such as holding the hand cannot be done via a camera [34]. Hence, eldercare personnel will always be needed; no technology can ever fully replace them. However, surveillance cameras can improve the work environment for employees, as well as the living environment for residents.

### Limitations of the study

One limitation of this study is that it does not explore the experiences of residents and relatives. The acceptance of digital surveillance cameras among residents and relatives has only been explored indirectly via the eldercare personnel. To get a deeper insight into how surveillance cameras are experienced by all stakeholders, it is important to ask them.

Another limitation is that we have not conducted any observations at the nursing homes. Therefore, we have not been able to directly observe the aspects that have emerged from this study.

The study mostly included staff working night shifts. Including more day-shift staff in the study could have yielded more varied results. Since many of the residents at the nursing homes had cognitive impairments to some degree, it was difficult to ask most of them to participate in the study. Therefore, we have not included any of the residents in this study.

## Conclusions

The aim of this paper was to analyze obstacles and opportunities associated with implementation and use of surveillance cameras at nursing homes from the perspectives of the eldercare personnel who use the technology, their working environments, and the conditions of the older people with cognitive impairment who live in nursing homes. The main themes that emerged from the study are the importance of prerequisites for implementation, improvements in the working environment at night, and possibilities to maintain security and integrity for the older people who live in nursing homes. An introductory education should be provided to the eldercare personnel in connection to the implementation, to get a smooth start of the intervention. Support from the supplier is also needed, and it is important that the support personnel have knowledge and own experience of using surveillance cameras. The functionality needs to be stable, and the start-up time should not be too long.

Surveillance cameras can make it easier for the eldercare personnel to prioritize and help each other across departments. Overall, cameras may contribute to making the work smoother. In addition, the eldercare personnel do not need to disturb the residents unnecessarily, when they have cameras. However, surveillance cameras cannot be a substitute to human hands and senses. Eldercare personnel cannot smell, feel the body temperature, see the face color, hear the breathing, and so on, through a camera.

This research provides insights into implementation and use of surveillance cameras as a welfare technology used at nursing homes. There is, however, a need for more research with regard to implementation and use of surveillance cameras as support in older people’s own private homes. Usually, eldercare personnel drive by car, sometimes several kilometers, when an older person needs support at home, or when an alarm has been activated. If surveillance cameras were used, some unnecessary visits to older people’s homes could be avoided. It is also important to study the perspectives of the older people and their relatives in more depth.

## Data Availability

All data generated or analyzed during this study are included in this published article. The data collected in this study are available from University West, but restrictions apply to the availability of these data according to the approval for this study by the Swedish Ethics Review Authority, ref. no. 932–18. Therefore, the data are not publicly available.

## Abbreviations

IP: Interview person
